# Preterm Labor Using Tocolysis as a Possible Risk Factor for Postpartum Depression: A 14-Year Population-Based Study in Taiwan

**DOI:** 10.3390/ijerph18137211

**Published:** 2021-07-05

**Authors:** Jui-Ming Liu, Chien-Yu Liu, Ren-Jun Hsu, Fung-Wei Chang

**Affiliations:** 1 Division of Urology, Department of Surgery, Taoyuan General Hospital, Ministry of Health and Welfare, Taoyuan 330, Taiwan; mento1218@gmail.com; 2 Department of Emergency Medicine, China Medical University Hospital, Taichung 404, Taiwan; zit.vincent@gmail.com; 3 Department of Bioinformatics and Medical Engineering, Asia University, Taichung 413, Taiwan; 4 Cancer Center, Hualien Tzu Chi Hospital, Buddhist Tzu Chi Medical Foundation, Hualien 970, Taiwan; 5 College of Medicine, Tzu Chi University, Hualien 970, Taiwan; 6 Department of Obstetrics & Gynecology, Tri-Service General Hospital, National Defense Medical Center, Taipei 114, Taiwan

**Keywords:** National Health Insurance Research Database, population-based study, postpartum depression, ritodrine

## Abstract

Postpartum depression (PPD) is associated with negative physical and mental health outcomes for the mother and infant. Women often experience elevated symptoms of PPD, and the incidence of PPD has increased in recent years. There were lack of studies to investigate the effects of medications during pregnancy. Herein, we focused on the most common obstetric medical therapies used in labor and determined whether the medical therapies cause mental stress in pregnant women. This 14-year retrospective population-based nationwide study was based on the National Health Insurance Research Database. Univariate and multivariate logistic regression analyses were used to evaluate unadjusted and adjusted odds ratios and 95% confidence intervals for each tocolytic and uterotonic treatments during pregnancy and common medical illnesses. In comparing the effects of tocolytic and uterotonic medications on maternal PPD, tocolysis with the injection form of ritodrine resulted in a significantly higher risk of PPD based on multivariate analysis. This study supports existing research demonstrating an association between tocolysis with ritodrine and PPD. Ritodrine treatment for preterm labor was a significant risk factor for PPD, especially the injection form. This information provides obstetricians and health policy providers to pay attention to maternal mental health outcomes among high-risk pregnant women.

## 1. Introduction

Postpartum depression (PPD) is an international public health priority. PPD is the most common cause of postnatal morbidity, with a prevalence as high as 19%, thus presenting a challenging target for prevention [1,2,3]. Postpartum mental health problems are recognized as a suitable target for preventive approaches, with the potential to avert the burden to women, their children and families, as well as the social and economic costs [4]. Women experience physical and mental changes during pregnancy and childbirth. These changes may cause a depressive mood. PPD is defined as a major depressive episode that begins during pregnancy or within 4 weeks after delivery [5]. PPD is widely reported worldwide with a prevalence from 0.5–60.8% [6,7]. Huang et al. [8] conducted a study, which showed a 19% and 18% prevalence of PPD in Taiwan and the United Kingdom, respectively. Many obstetric and pediatric factors are associated with PPD, including unplanned pregnancy, parity, newborns with medical illnesses, preterm labor, and complications during pregnancy [6,9,10,11,12,13]. Some medical illnesses affecting the fetus, infant, and pregnant women need medications for treatment. Medications for these illnesses may cause mental stress on pregnant women [14].

The preterm birth rate in the United States had declined from 12.8% in 2006 to 11.7% in 2011, especially in the late-preterm category (34–36 weeks) [15]. In undeveloped and developing countries, the preterm birth rate was around 6% to 10% of all births, leading an important cause of perinatal mortality and morbidity [16]. In Taiwan, the preterm birth rate was around 8.56% of those who delivered between 32 and 37 weeks of gestation [17].

The perinatal morbidity was necrotizing enterocolitis, intracranial bleeding, and respiratory distress syndrome, and the neonatal mortalities were approximately two-thirds. It caused serious social and economic losses [18,19,20]. Therefore, it is important to improve perinatal outcomes by using maternal tocolytic treatments to prevent or delay preterm births. The U.S. Food and Drug Administration (FDA) had approved the beta-adrenergic receptor agonist ritodrine for preterm labor. Parenteral ritodrine confirmed a delivery delay up to 48 hours. The delay may aid maternal transport to tertiary care or permit fetal lung maturation with corticosteroids. The maternal effects and side effects of ritodrine are well known; pulmonary edema is a special concern. However, the possible adverse mental effects on pregnant women remain relatively unknown. In this present study, we have determined the clinical mental side effects of ritodrine, the most frequently used tocolytic agent, and evaluated the effects on PPD.

Oxytocin, as a uterotonic agent, is central to normal birth, lactation, and mother–infant attachment [20,21]. Oxytocin also plays a key role in regulating emotion, social interaction, and stress reactivity [22,23]. In addition, reductions in oxytocin measured in plasma [24,25] have been associated with PPD. Synthetic oxytocin is commonly used in labor management to induce and augment labor and to prevent postpartum hemorrhage. Higher synthetic oxytocin doses are associated with increased depressive, anxious, and somatization symptoms [26]. Inconsistencies in the reports of an association of PPD may depend on endogenous or synthetic oxytocin. The long-term consequences of oxytocin for maternal mental health and behavior are largely understudied. We determined the relationship between intrapartum synthetic oxytocin and postpartum maternal mental health.

Women often experience elevated symptoms of PPD, and the incidence of PPD has increased in recent years. There is also a lack of studies to investigate the effects of medications during pregnancy. Herein, we have focused on two of the most common obstetric medical therapies used in labor (tocolytics and uterotonic) and determined whether or not the medical therapies cause mental stress in pregnant women. Our secondary objective was to investigate the mental side effects of medications. Thus, we conducted a 14-year nationwide population-based study from 2000 to 2013 to determine the relationship between medications during pregnancy and PPD.

## 2. Materials and Methods

### 2.1. Data Source and Collection

This population-based study used the National Health Insurance (NHI) database. The NHI database covers 99.9% of the 23 million residents in Taiwan. The Taiwan NHI program began in 1995. The NHI database contains nationwide medical record and demographic information for inpatients and outpatients in Taiwan [27,28]

We performed a retrospective study retrieved from the Longitudinal Health Insurance Database 2000 (LHID2000), a sub-dataset of the NHI research database (NHIRD), between January 2000 and December 2013. The LHID2000 randomly selected 1 million residents of the 23 million residents included in the NHIRD in 2000. The demographic distribution of residents in the LHID2000 and the original population in the NHIRD are similar [29]. The disease diagnosis was based on the International Classification of Diseases, 9th revision, Clinical Modification (ICD-9-CM) [30]. This retrospective study was approved by the Institutional Review Board of the Tri-Service General Hospital (approval no. B-104-20, Taipei, Taiwan), and the requirement to obtain informed consent was waived.

### 2.2. Study Population

**Figure 1:**
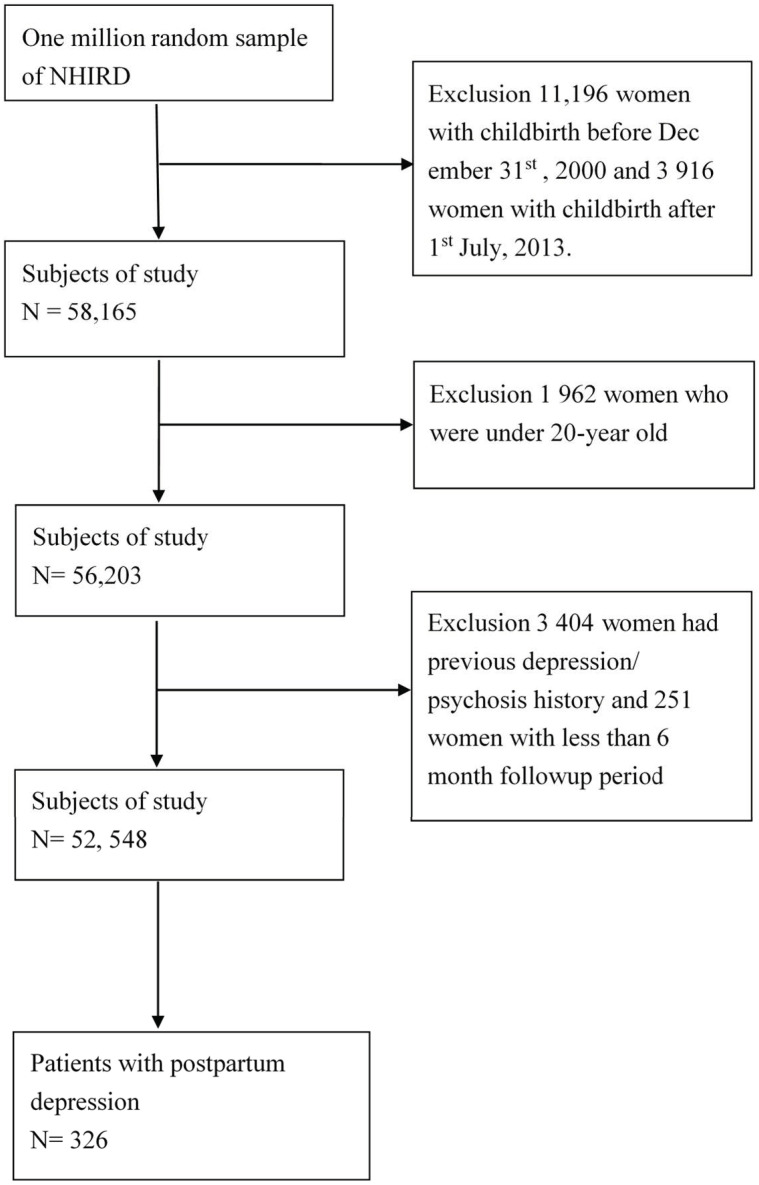
Flowchart of recruitment of subjects from the 1 million random sample of the National Health Insurance Research Database (NHIRD) from 2000 to 2013 in Taiwan.

The study subjects were selected from the LHID2000 database between January 2000 and December 2013. Figure 1 shows the selection process for the study population. We first selected females whose first delivery was between January 2000 and December 2013. The exclusion criteria included the following: (1) primiparas with a delivery date before 31 December 2000 or after 1 July 2013 (n = 15,112); (2) <20 years of age (n = 1962); (3) previous major depressive disorder or a history of psychosis (n = 3404); (4) follow-up period < 6 months (n = 251). Finally, we selected females who were newly diagnosed with major depressive disorder during the first 6 months after delivery (ICD-9-CM: 309, 311, 296.2, 296.3, 296.5, and 300.4). The diagnosis of major depressive disorder was established by psychiatrists who also provided treatment with antidepressants. The Charlson comorbidity index (CCI) score was used to evaluate the clinical status of the subjects. The CCI score contains 19 comorbid conditions based on the method of Quan et al. [30].

### 2.3. Covariates

Covariates were chosen in the current study based on the extant literature and clinical manifestations during pregnancy. We selected the following covariates: chronic pulmonary disease (ICD-9-CM: 490–492, 494, and 496); hypertension (ICD-9-CM: 401.1 and 401.9); hypertension-complicated pregnancy (ICD-9-CM: 642.0, 642.1, 642.2, 642.3, and 642.9); diabetes complicating pregnancy childbirth (ICD-9-CM: 648.0); hyperlipidemia (ICD-9-CM: 272.4); heart disease (ICD-9-CM: 393–398, 402, 404.0, 404.1, 404.9, 410–414, 415.0, 416.1, 416.8, 416.9, and 420–429); anemia (ICD-9- CM: 280–285); peptic ulcer disease (ICD-9-CM: 533); cerebrovascular disease (ICD-9-CM: 430–438); Parkinson’s disease (ICD-9-CM: 332); epilepsy (ICD-9-CM: 345); tuberculosis (ICD-9-CM: 011 and 012); asthma (ICD-9-CM: 493); liver cirrhosis (ICD-9-CM: 571); chronic kidney disease (ICD-9-CM: 585, 586, and 588); herpes (ICD-9-CM: 053 and 054); syphilis (ICD-9-CM: 090–097); antepartum hemorrhage (ICD-9-CM: 641.8 and 641.9); eclampsia or pre-eclampsia (ICD-9-CM: 642.4, 642.5, and 642.6); premature separation of placenta (ICD-9-CM: 641.2); placenta previa (ICD-9-CM: 641.0 and 641.1); unspecified disorder of the thyroid (ICD-9-CM: 46.9); unstable lie (ICD-9-CM: 652.0); oligohydramnios (ICD-9-CM: 658); polyhydramnios (ICD-9-CM: 657); poor fetal growth (ICD-9-CM: 656.5); excessive fetal growth (ICD-9-CM: 656.6); cervical incompetence (ICD-9-CM: 654.5); early delivery onset (ICD-9-CM: 644); other known or suspected fetal abnormality (ICD-9-CM: 655.8).

The monthly income of subjects based on insurance data of NHI was also included.

In addition, we chose two of the most common drug therapies during labor, which have a substantial influence on the mental status of women (tocolytics and uterotonics).

### 2.4. Statistical Analysis

Statistical analysis was performed by Microsoft SQL Server 2008 and IBM SPSS statistics software v20 (IBM SPSS, 2013). All medical records and baseline data were managed with Microsoft SQL Server 2008 and statistically analyzed using IBM SPSS statistics software v20 (IBM SPSS, 2013). Multivariate logistic regression was used to analyze the correlation between comorbidities, drug therapy during labor, and PPD. A two-sided *p* value < 0.05 was regarded as statistically significant.

## 3. Results

### 3.1. Demographics

A total of 52,548 primiparas were enrolled in this study between January 2001 and June 2013. There were 326 women with newly diagnosed PPD within 6 months after delivery during the study period. We divided the 52,548 primiparas into two groups, as follows: PPD (n = 326) and non-PPD (n = 52,222). The demographic characteristics for the study groups are shown in Table 1. There were no differences in age and hospital stay in the two groups. The average age of women with PPD was 29.5 ± 4.6 years, and the average length of hospital stay was 3.8 ± 2.6 days. Women with PPD had significantly elevated CCI scores compared with women without PPD (*p* = 0.03, 0.2 ± 0.5 vs. 0.1 ± 0.4). Some comorbidities were significantly increased in the PPD group, including hypertension (*p* = 0.01), peptic ulcer disease (*p* = 0.0008), chronic kidney disease (*p* = 0.004), heart disease (*p* < 0.001), unstable lie (*p* = 0.006), and early delivery onset (*p* < 0.001). Monthly incomes were significantly different between the two groups. Tocolytic medications were significantly associated with PPD (*p* = 0.002). Both the oral and injection forms of tocolytic medications were associated with PPD. In contrast, uterotonic medications were not associated with PPD.

### 3.2. Multivariable Analysis of Postpartum Depression

Table 2 reveals the logistic regression analysis for specific risk factors. No medications during pregnancy and the monthly income < NTD 20,000 were the reference. Chronic kidney disease (*p* = 0.007, odds ratio (OR) = 7.665, 95% confidence intervals (CI) = 1.749–33.595), heart disease (*p* = 0.002, OR = 2.302, 95% CI = 1.361–3.895), and unstable lie (*p* = 0.01, OR = 1.724, 95% CI: = 1.141–2.605) were significant risk factors of PPD. The OR of the monthly income > NTD 60,000, NTD 40,000–60,000, and NTD 20,000–40,000 was 0.487 (*p* = 0.005, 95% CI = 0.294–0.808), 0.656 (*p* = 0.026, 95% CI = 0.451–0.952), and 0.664 (*p* = 0.001, 95% CI = 0.523–0.843). The use of the injection form of tocolysis (*p* = 0.022, OR = 2.238, 95% CI = 1.122–4.463) was significantly associated with PPD. In contrast, uterotonic and oral tocolytic medications had no correlation with PPD.

## 4. Discussion

Pregnant women are easily affected by physical, mental, or surrounding changes that cause a depressed mood. According to the fifth edition of the Diagnostic and Statistical Manual of Mental Disorders (DSM-5) [31], PPD is defined as a major depressive episode occurring during pregnancy or in the 4 weeks following delivery. This is the first study involving medications during pregnancy and PPD using a national population-based method. In the present study, it was determined that ritodrine, when used as a tocolytic agent in preterm labor, was associated with PPD. In particular, the injection form of ritodrine (*p* = 0.022, OR = 2.238, 95% CI = 1.122–4.463) was a significant risk factor for PPD after logistic regression analysis. In addition, chronic kidney disease, heart disease, an unstable lie, and lower monthly income were significant risk factors for PPD. Our results provide useful information for physicians and pregnant women with respect to the early prevention of PPD.

Based on univariate analysis, tocolytic medications were significantly associated with PPD. Pregnant women who require tocolytic medications are at risk for preterm labor. Preterm labor is a strong risk factor for PPD [32,33]. Adewuya et al. [34] reported that Nigerian women with preterm labor had 4.21 times the risk for PPD (OR = 4.21, CI = 2.78–6.39). Drewett et al. [35] concluded that women with preterm labor had 1.6 times the risk of PPD 8 weeks thereafter (adjusted relative risk = 1.6, 95% CI = 1.2–2.1). Logistic regression analysis showed that the injection form of tocolytic medications had 2.238 times the risk for PPD in the current study (OR = 2.238, 95% CI = 1.122–4.463). We suggest that pregnant women who require the injection form of tocolytic medications are under stress caused by the physical, mental, and surrounding changes leading to the development of PPD. In the current study, the oral form of tocolytic medications was not a risk factor for PPD based on logistic regression analysis. The injection versus oral forms of tocolytic medications are used depending on the severity of preterm labor. Indeed, a low probability of preterm labor only requires an oral form of tocolytic medication, hydration, and bed rest at home. In such cases, the stress associated with physical, mental, or surrounding changes is not sufficiently severe to affect the pregnant woman’s mood. Different degrees of severity of preterm labor induces different stress on women with pregnancy. Vigod et al. [32] published a review article that showed women with preterm labor are at higher risk for PPD than women with term labor. Vigod et al. [32] also showed that women with very preterm labor had a continued risk of PPD throughout the first postpartum year. In addition, women with preterm labor have greater opportunity to receive cesarean sections, which are associated with PPD [36,37,38,39].

Uterotonic drugs, such as synthetic oxytocin, had no significant correlation with postpartum anxiety or PPD in the current study. Andersson et al. [40] reported that post-term labor had no correlation with postpartum anxiety or PPD, and oxytocin use had no significant association with postpartum anxiety or PDD. Nevertheless, higher synthetic oxytocin dose was associated with greater depressive, anxious, and somatization symptoms [26]. The inconsistencies in the reports of association of PPD may depend on ethnicity, background, the nature of behaviors involved, social salience, social environment, and individual social factors. In addition, post-term infants are usually healthier than preterm infants and imposed little mental stress on women. The induction of post-term infants leads to fewer neonatal deaths or complications [41].

This study showed that heart disease, an unstable lie, lower monthly income, and chronic kidney disease are significant risk factors for PPD. Yang et al. [36] also showed that heart disease is associated with PPD, but unstable lie is not. An unstable lie may need additional time for labor with an increased use of epidural analgesia. Chung et al. [42] reported that epidural analgesia is related to PPD. Several studies support that low income is associated with PPD [43,44]. Chronic kidney disease, such as kidney and urinary infections, might be associated with PPD symptoms and a PPD diagnosis [45].

Finally, it is obligatory to emphasize the situation of tocolytic agents, which is important for preterm labor, while discussing the results of our study on the effects of ritodrine on maternal PPD. There were several limitations to this study. This was a retrospective population-based study. Some data, such as the marital relationship, education status, and employment status, were not available in the NHIRD database, and these data are also important risk factors for PPD. The selected time interval of our study is limited due to the LHID2000 database being between January 2000 and December 2013, which could not be updated and did not contain the latest information. In the current study, we had 14-year study periods, and the study results were able to provide useful information. The diagnosis of PPD was made by psychiatrists in this study. It is evitable that every psychiatrist may have individual judgements, even under DSM V guidelines. Women who experienced mental stress might not consult psychiatrists; thus, PPD is underestimated. The number of patients was relatively small due to our strict inclusion criteria (primiparas only). We only chose two kinds of obstetric medications for the investigation. There are still other medications worthy of study. Further prospective studies are needed to evaluate the relationship between drug therapy during labor and PPD.

## 5. Conclusions

Although we know a lot about the pharmacokinetics and pharmacodynamics of these drugs, there are no data about their effects on maternal mental health. Tocolytic drugs are a significant risk factor for PPD, especially the injection form. In contrast, uterotonic drugs had no correlation with PPD. This result indicates that preterm labor causes much more mental stress on women than post-term labor. In addition, chronic kidney disease, heart disease, unstable lie, and low monthly income were also risk factors for PPD. Further prospective research to investigate the relationship between drug therapy during labor and PPD is recommended. We have provided useful information for gynecologists, obstetricians, and health policy providers that should pay attention to the influence of drug therapy during labor on mental stress.

## Figures and Tables

**Table 1:** Demographic characteristics associated with the subjects in Taiwan, 2000 to 2013.

Demographic Factor	Total Pregnant Women (%)	Women without PPD (%)	Women with PPD (%)	*p* Value ^a^
No. of patients	52,548	52,222	326	
Mean age (standard deviation) Age (mean ± SD, years)	29.6 ± 4.6	29.6 ± 4.6	29.5 ± 4.6	0.853
20–24	8747 (16.6)	8688(16.6)	59(18.1)	
25–29	20,149(38.4)	20,027(38.4)	122(37.5)	
30–34	17,205(32.7)	17,101(32.7)	104(31.9)	
35–39	5513(10.5)	5479(10.5)	34(10.4)	
≥40	934(1.8)	927(1.8)	7(2.1)	
Charlson comorbidity index	0.1 ± 0.4	0.1 ± 0.4	0.2 ± 0.5	0.031
0	47,243(89.9)	46,963(89.9)	280(85.9)	
1	4597(8.7)	4560(8.7)	37(11.4)	
2	562(1.1)	555(1.1)	7(2.1)	
≥3	146(0.3)	144(0.3)	2(0.6)	
Comorbidity disease				
Chronic pulmonary disease	1507(2.9)	1495(2.9)	12(3.7)	0.401
Hypertension	475(0.9)	467(0.9)	8(2.5)	0.010
Hypertension-complicated pregnancy	811(1.5)	802(1.5)	9(2.8)	0.106
Diabetes complicating pregnancy childbirth	2542(4.8)	2527(4.8)	15(4.6)	0.999
Hyperlipidemia	135(0.3)	134(0.3)	1(0.3)	0.569
Heart disease	976(1.9)	960(1.8)	16(4.9)	<0.001
Anemia	3644(6.2)	3624(6.2)	20(6.1)	0.987
Peptic ulcer disease	1743(3.3)	1723(3.3)	20(6.1)	0.008
Cerebrovascular disease	55(0.1)	55(0.1)	0 (0)	1.00
Parkinson’s disease	2(0.1)	2(0.1)	2(0)	1.00
Epilepsy	62(0.1)	62(0.1)	0(0)	1.000
Tuberculosis	33(0.1)	33(0.1)	0(0)	1.00
Asthma	605(1.2)	602(1.2)	3(0.9)	0.998
Liver cirrhosis	822(1.6)	814(1.6)	8(2.5)	0.178
Chronic kidney disease	51(0.1)	48(0.1)	3(0.9)	0.004
Herpes	853(1.6)	845(1.6)	8(2.5)	0.263
Syphilis	75(0.1)	74(0.1)	1(0.3)	0.373
Antepartum hemorrhage	3739(7.1)	3712(7.1)	27(8.3)	0.392
Premature separation of placenta	153(0.3)	151(0.3)	2(0.6)	0.245
Placenta previa	1017(1.9)	1008(1.9)	9(2.8)	0.306
Eclampsia or pre-eclampsia	539(1.0)	533(1.0)	6(1.8)	0.155
Unspecified disorder of the thyroid	2(0.1)	2(0.1)	0(0)	1.000
Unstable lie	2296(4.4)	2271(4.3)	65(7.7)	0.006
Polyhydramnios	214(0.4)	214(0.4)	0(0)	1.000
Oligohydramnios	1326(2.5)	1314(2.5)	14(3.7)	0.209
Poor fetal growth	1905(3.6)	1899(3.6)	16(4.9)	0.231
Excessive fetal growth	985(2.9)	980(2.9)	5(3.7)	0.838
Other known or suspected fetal abnormality	147(0.3)	145(0.3)	2(0.6)	0.232
Cervical incompetence	127(0.2)	125(0.2)	2(0.6)	0.187
Early delivery onset	12,959 (24.7)	12,846 (24.6)	113(34.7)	<0.001
Hospital stay (mean ± SD, days)	3.6 ± 1.6	3.6 ± 1.6	3.8 ± 2.6	0.128
Monthly income (TWD ^b^)				0.001
<20,000	17,012(32.4)	16,873(32.4)	139(42.6)	
20,000–40,000	24,733(47.1)	24,598(47.1)	135(42.4)	
40,000–60,000	6504(12.4)	6469(12.4)	35(10.7)	
≥60,000	4299(8.1)	4282(8.1)	17(5.3)	
Tocolytic drugs				0.002
Yes	3266(6.2)	3232(6.2)	34(10.4)	
Only oral form	2220(4.2)	2197(4.2)	23(7.1)	0.018
Only injection form	500(1.0)	491(1.0)	9(2.7)	0.004
Both oral and injection forms	546(1.0)	544(1.0)	2(0.6)	0.780
No	492,824(93.8)	48,990(93.8)	292(89.6)	
Uterotonic drugs				0.178
Yes	37,228(70.8)	37,008(70.9)	220(67.5)	
No	15,320(29.2)	15,214(79.1)	106(32.5)	

PPD, postpartum depression; SD, standard deviation; ^a^. *p*-values are two-sided. ^b^ TWD refers to New Taiwan dollars, of which 1 US dollar = 32 TWD.

**Table 2:** Multivariate analyses of certain risk factors of PPD.

	Estimate	Standard Error	Wals Chi-Square	Adjusted Odds Ratio	95% Confidence Interval	*p* Value
Charlson comorbidity index (reference: 0)						
1	0.047	0.224	0.044	1.048	(0.676–1.626)	0.834
2	0.117	0.470	0.062	1.124	(0.447–2.827)	0.804
≥3	−0.225	0.827	0.074	0.798	(0.158–4.040)	0.786
Hypertension	0.692	0.378	3.345	1.998	(0.952–4.194)	0.067
Peptic ulcer disease	0.547	0.307	3.183	1.728	(0.947–3.153)	0.074
Chronic kidney disease	2.037	0.754	7.298	7.665	(1.749–33.595)	0.007
Heart disease	0.834	0.268	9.665	2.302	(1.361–3.895)	0.002
Unstable lie	0.545	0.211	6.676	1.724	(1.141–2.605)	0.010
Early delivery onset	0.253	0.454	0.311	1.288	(0.529–3.138)	0.577
Monthly income (reference: <20,000)						
20,000–40,000	−0.409	0.121	11.343	0.664	(0.524–0.843)	0.001
40,000–60,000	−0.416	0.190	4.785	0.659	(0.454–0.958)	0.029
≥60,000	−0.713	0.258	7.624	0.490	(0.295–0.813)	0.006
Premature birth	0.141	0.455	0.096	1.151	(0.471–2.881)	0.757
Tocolytic drugs (reference: no)						
Only oral form	0.340	0.228	2.235	1.405	(0.900–2.196)	0.135
Only injection form	0.806	0.352	5.232	2.238	(1.122–4.463)	0.022
Both oral and injection forms	−0.779	0.717	1.181	0.459	(0.113–1.870)	0.277
Uterotonic drugs	−0.180	0.120	2.260	0.835	(0.660–1.056)	0.133

## Data Availability

The authors’ are unable to share their data because the Taiwan National Health Insurance Research Database data can only be prescribed to applicants.

## Abbreviations

PPD = postpartum depression, ICD-9-CM = International Classification of Diseases, 9th Revision, Clinical Modification, NHIRD = National Health Insurance Research Database, LHID2000 = Longitudinal Health Insurance Database2000, CCI = Charlson comorbidity index, OR = odds ratio.
